# Bio-Liquid Morphological Analysis

**DOI:** 10.1100/tsw.2004.118

**Published:** 2004-08-18

**Authors:** S.N. Shatokhina, V.N. Shabalin, M.E. Buzoverya, V.T. Punin

**Affiliations:** ^1^ Scientific-Research Institute of Gerontology, Ministry of Health, Russian Federation; ^2^ Russian Federal Nuclear Center, All-Russia Scientific-Research Institute of Experimental Physics, Russian Federation

**Keywords:** optical microscopy, bio-liquid morphological analysis, new diagnostic technologies, structure quantitative processing, protein self-organization

## Abstract

Information is presented on the new scientific line in medicine and biology: bio-liquid morphology. The interdisciplinary character of the given research area is emphasized. The problems and prospects of bio-liquid morphological analysis development both in applied and fundamental aspects are discussed.

Structure, being the most important quality of any material object, on one hand mirrors the conditions of its formation and on the other hand determines its physical and operational properties. Therefore, considerable attention is paid to structure in various areas of science, including biology and medicine.

Until recently in medical practice, the biological tissue structure examination was applied only to cellular and higher forms of its organization. Genetically determinate cellular forms of living systems are well known. They are studied within frameworks of such disciplines as anatomy, histology, cytology, etc. Structures of bio-liquids for medicine, practical medicine in particular, are still not adequately investigated. The concept of structure has been considered to be inapplicable to biological liquids because of the extremely high variability of their molecular composition and the character of the components interaction. Along with this, at the molecular and permolecular levels, the bio-liquids possess determinate, rather stable orderliness.

In the 1990s in Russia, a new scientific line in medicine and biology was formed: bio-liquid morphology[1]. It is methodically based on the biological liquid being brought into solid phase with a following examination with an optical microscope. The obtained data contain information not only of the concentration of elements — constituents of such complex system — but of the character of their interrelation as well. Morphological parameters of the biological liquids have distinct peculiarities and are used as the diagnostic criteria for determining diverse pathological conditions in different organs and systems, homeostasis stability degree, biological age, and the applied therapy efficiency. At present, the given scientific line is widely introduced in medicine and is already applied in therapy, surgery, pediatrics, urology, etc.[2,3,4,5]. Research in the area of biological liquid morphology is not only of practical importance, but also discloses principally novel approaches in the living matter organization study, and is of interest not for clinicians, biologists, and biophysicists only, but for investigators in the area of complex system behavior and self-organization as well.

Bio-liquids participate in all processes of intra- and extracellular metabolism. Among the bio-liquids are blood serum, lymph, cerebrospinal fluid, secretions of endocrine and exocrine glands, urine, etc. Biochemical, physical-chemical, immunological, and other methods of bio-liquid examination give fragmentary data on the body's condition, while the structural analysis allows information of the system as a whole to be obtained. To extract such information, a special method has been developed of bio-liquid dehydration with preset angular profile of the mass being dried — cuneiform dehydration method. At cuneiform dehydration, the molecules and permolecular complexes are distributed in strictly defined places of the drying drop surface in the form of concentration zones. As a result, a **facies** is formed, the structure-specific character of which is determined by the entire set of qualitative and quantitative parameters of the substances present in the bio-liquid (Fig. 1). That is, the bio-liquid dehydrated drop is a standard thin slice of highly mobile tissue, the structures of which are being ordered and fixed during the dehydration process.

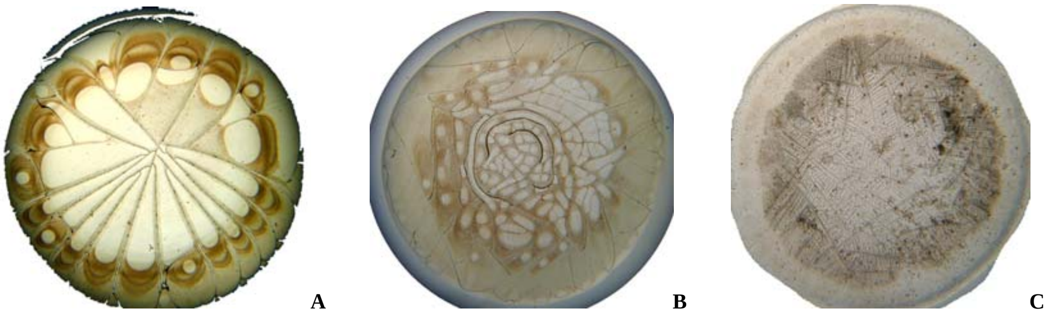

A B C

**FIGURE 1.** Facies: (A) blood serum at norm, (B) blood serum at pathology, (C) saliva at pathology.

The facies appearance is used now to diagnose a wide circle of diseases. Pathological changes that take place in the body lead to violation of the chemical activity of its structures, which is reflected in morphology of the bio-liquid solid phase.

For the blood serum facies of a healthy man, symmetry of the cracks allocation is typical. The cracks are radially oriented, positioned throughout the entire facies area, and divided into radial sectors approximately equal in size, in which the concretions are situated (Fig. 1A).

Pathological states are characterized by an asymmetrical disposition of cracks, which, in their turn, form irregularly located sectors and singularities (Fig. 1B). The structure of facies of biological liquids with small protein content and increased salt content (saliva, urine, etc) differs considerably from the blood serum structure. Facies of such bio-liquids have two areas: a peripheral area shaped as a protein ring and a central area filled with salt dendrites (Fig. 1C).

Today, markers of sclerotic changes, inflammatory processes, and other pathological body conditions have been established. The obtained results of the morphologic analysis show good agreement with the clinical data of the examined persons' health, and the biochemical and immunological tests.

In studying a material structure, the exploration of structural peculiarities of its separate elements is of great importance. In terms of spatial microstructure geometry, any material could be conceived as a conglomerate, consisting of a set of microscopic bodies filling a certain space and connected with each other by the contact surfaces (microparticles). They are structural units of a microscopic structure, much like the elementary cell is a structural unit of crystalline structure. Among the structural elements of bio-liquid facies are cracks (Fig. 2A), concretions (Fig. 2A), dendrites (Fig. 2B), foreign inclusions (Fig. 2C), etc.

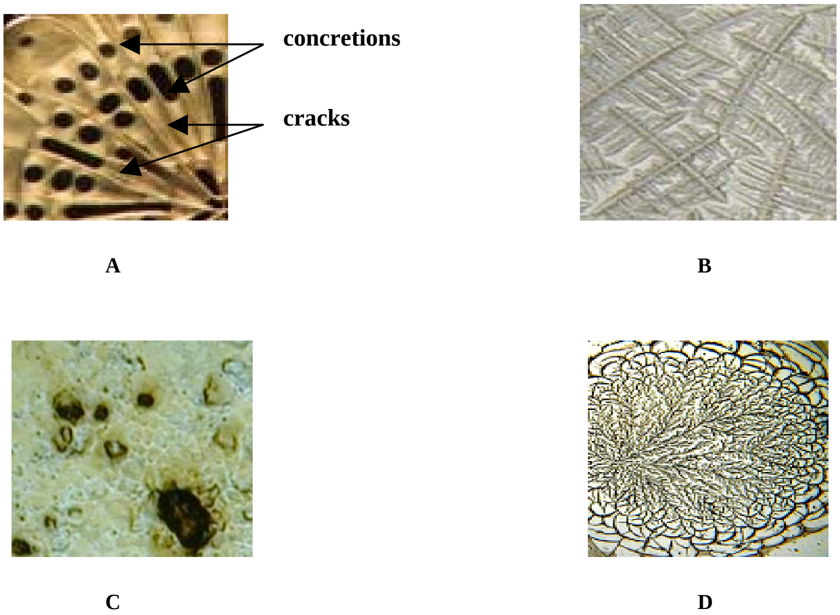

**FIGURE 2**. Structural elements of the bio-liquids. (A) System of cracks and concretions, (B) dendrites, (C) inclusions, (D) fractals.

At present, to analyze bio-liquid structure, the descriptive approach is used. Quantitative estimation of the dehydrated bio-liquid structure makes it possible to involve the effective mathematic device as well as the computer technique to find analytical dependences, which will enable us to reveal the nature of the physical-chemical processes that take place in the material. In addition, quantitative description of the bio-liquid morphological parameters will allow new diagnostic tests to be developed.

When studying the bio-structural effects, we noticed some analogies in the character of aging and cracking processes in polymers and bio-liquids**.** This made it possible to use the mathematic device developed for solid-object, multiple-fracture processes describing for structures of blood serum and protein preparations objectivization[6].

In the course of investigations, the hardware-software system “Morphotest” was developed[7]. The computer-aided system of biological liquid structure analysis allows facies images to be obtained, digitized, recognized, and analyzed. The possibility of photographs, files, and assays evolving into analysis essentially widens the system capabilities. Special software methods of image improvement, original techniques to reveal diagnostically valuable structural elements, allow facies qualitative characteristics to be obtained.

A “Morphology” database has been created. It is constantly populated by different types of pathologies and more precise quantitative criteria are elaborated.

*The method enables the effects to be visualized and studied, which earlier could not be observed[8]*. Now high sensibility of living organisms to weak physical fields is of interest (small radiation doses, low-intensity electro-magnetic fields, laser radiation). That is why the task of detecting these reactions and studying the mechanisms of their transformation into biological functions now appears. The bio-liquid could be considered as an indicator of weak physical signals. The bio-liquid provides a simple model, useful and convenient for qualitatively and quantitatively describing certain cooperative processes that take place at a molecular and permolecular level under the influence of low-intensity factors.

The blood serum compound composition, along with the diversity of the body’s physical-chemical processes, makes it difficult to decide what specific factors result in formation of those particular structures observed in the facies. In order to reveal the causes leading to some particular pathological structures formation, we are investigating protein artificial aging using simple models (albumin, in particular)[9]. The use of model liquids of simple composition allows us to reduce to a minimum the number of factors affecting the structure formation, as well as to provide the varying liquid parameters controlled. The present researches, in the authors' opinion, are to stimulate investigations of the self-organization processes in protein — basic component of the living matter.

At present, a large amount of experimental data on the bio-liquid structures has been accumulated. The data classification, explanation in unified terms, and search for new directions for further research are the main tasks of this novel scientific line development.

In conclusion, we would like to note that the body's bio-liquids, as an object to be studied, are of interest for specialists of several scientific lines, because this is a problem of great scientific interest and practical potentialities. The task of establishing the mechanisms of bio-liquid structure formation is one of the most complicated problems of physical-chemical hydrodynamics, theory of heat and mass transfer, and phase changes. It is a part of the general scientific problem of isotropy, order, and chaos relations (self-organization).

## ACKNOWLEDGMENTS

The authors would like to express their gratitude to Professor Margaret Tzaphlidou, TheScientificWorldJOURNAL Editor, for giving them a chance to present the results of their research to the international scientific community and for handling the manuscript. The authors are grateful to S.N. Abramovich and Yu.P. Sherbak for their assistance in carrying out the research and discussing the obtained results, and for their valuable remarks made during the discussion.

## References

1. Shabalin, V.N. and Shatokhina, S.N. (2001) *Human Bio-Liquids Morphology*. Chrizostom, Moscow. p. 304.
2. Zaytsev, V.V. (1996) Textures of biological liquid crystals in patients with cardiac infarction. *Proc. Acad. Sci.* **60(4),** 115–118.
3. Rapis, E.G. (2002) Change of physical phase of the of blood serum protein complex non-equilibrium film in patients with carcinoma. *Tech. Phys. J.* **72(4),** 139–142.
4. Buzoverya, M.E., Punin, V.T., and Abramovich, S.N. (2002) Proton microprobe technique in element analysis of biological liquids. *Metal Ions Biol. Med*. **7,** 21–25.
5. Potekhina, Yu.P., Zubeev, P.S., et al. (2000) Crystallographic analysis of biological liquids at cholelithiasis. *Int. Med. J.* **5**, 469–472.
6. Buzoverya, M.E., Selchenkova, N.I., Uchaev, A.Ya., Shabalin, V.N., and Shatokhina, S.N. (2001) About fractal properties of inhomogeneities and microcracs arising at blood serum dehydration. Proceedings of the 4th International Conference on Mathematical Models of Non-Linear Excitations, Transfer, Dynamics, Control in Condensed Systems and Other Media, Moscow. pp. 184–193.
7. Buzoverya, M.E., Shabalin, V.N., et al. (2003) Software Package "Morphotest" for Bio-Liquid Morphological Analysis. Certificate #2003612503 of 13.11.03.
8. Buzoverya, M.E., Selchenkov, V.L., Sherbak, Yu.P., et al. (2002) Bio-liquid morphological analysis for studying therapeutic effects of low-frequency electromagnetic fields. *Biomed. Technol. Radioelectron*. **2**, 49–52.
9. Buzoverya, M.E. and Shishpor, I.V. (2003) Study of the oxidative destruction influence on albumin morphology. Collected Theses of Reports of the 12th International Conference on Flows and Structures in Liquids, St. Petersburg. 2003.

## BIOSKETCHES

**Shatokhina Svetlana Nikolaevna** — Doctor of Medicine, Head of the Laboratory of Bio-liquid Morphology of the Scientific-Research Institute of Gerontology of the Ministry of Health of the Russian Federation. Past and current research interests: clinical laboratory diagnostics, hematology, cytology, bio-liquid morphology.

**Shabalin Vladimir Nikolaevich** — Professor, Academician of the Russian Academy of Science, Director of the Scientific-Research Institute of Gerontology of the Ministry of Health of the Russian Federation. Past and current research interests: immunology, genetics, gerontology, bio-liquid morphology.

**Buzoverya Marina Eduardovna** — Senior Staff Scientist of the Russian Federal Nuclear Center, All-Russian Scientific-Research Institute of Experimental Physics. Past and current research interests: material technology, methods of physical-chemical analysis, polymer (bio-polymer) aging.

**Punin Vitaly Tikhonovich** — Doctor of Science, Deputy Scientific Manager of the Russian Federal Nuclear Center, All-Russian Scientific-Research Institute of Experimental Physics. Past and current research interests: thermo-nuclear fusion, nuclear physics, medicine physics.
